# Shoulder pain and functional disability in type 1 diabetic patients: A cross-sectional survey

**DOI:** 10.12669/pjms.37.4.3401

**Published:** 2021

**Authors:** Muhammad Nazim Farooq, Aqsa Mehmood, Fatima Amjad, Jaweria Syed

**Affiliations:** 1 Muhammad Nazim Farooq, PhD. Islamabad College of Physiotherapy, Margalla Institute of Health Sciences Rawalpindi, Rawalpindi, Pakistan; 2 Aqsa Mehmood, DPT Islamabad College of Physiotherapy, Margalla Institute of Health Sciences Rawalpindi, Rawalpindi, Pakistan; 3 Fatima Amjad, DPT Islamabad College of Physiotherapy, Margalla Institute of Health Sciences Rawalpindi, Rawalpindi, Pakistan; 4 Jaweria Syed, MS-SPT Shifa Tameer-e-Millat University, Islamabad, Pakistan

**Keywords:** Diabetes mellitus, Type-1, Prevalence, Risk factors, Shoulder pain

## Abstract

**Objective:**

To assess the prevalence of shoulder pain and functional disability (SPFD) in Type-1 diabetic patients, and to explore its association with duration of the disease, age and gender.

**Methods:**

A cross-sectional survey was carried out on previously diagnosed patients with Type-1 diabetes mellitus between April 2019 and March 2020. Data was collected from six hospitals including three tertiary care hospitals of Islamabad and Rawalpindi. Three hundred and twenty-eight patients were recruited through convenience sampling. Shoulder Pain and Disability Index was used to determine SPFD among participants. Point-biserial and Pearson correlation coefficients were calculated to find out the correlation between the variables. Independent t-test was used to determine the difference in the mean scores between the variables.

**Results:**

The prevalence of SPFD was found 85.7%. A significant correlation was found of the SPFD with age (r = 0.332, p < 0.001), duration of the diabetes mellitus (r = 0.154, p = 0.005) and gender (r_pb_ = 0.171, p = 0.002). A significant difference was found in SPFD mean scores between female and male patients (female patients = 43.42±22.80, male patients = 35.31±22.91, p = 0.002).

**Conclusion:**

SPFD seems prevalent among Type-1 diabetic patients. Increasing age, long history of diabetes mellitus and female gender appear the associated risk factors for the shoulder pain and disability.

## INTRODUCTION

Diabetes mellitus (DM) is a metabolic disorder characterized by hyperglycemia. It may be either due to decline/absence of production of insulin by pancreas or when pancreas produces enough insulin but the body cannot utilize it or due to both.^1^ DM is a major health problem touching alarming level. The number of people with DM has been increased by 62% during the last decade worldwide.^2^ Globally 463 million people were estimated to have DM in 2019.^2^ The Pakistan was at fourth number among the top ten countries having highest number of people with DM. The diabetic population is continuously increasing in Pakistan.^3^ where 19.4 million people were estimated to have DM in 2019 which will rise to 26.2 million in 2030 and 37.1 million in 2045.^2^ Type-1 DM accounts for about 5–15% of all diabetes cases.^4^ It results from an autoimmune destruction of insulin-producing β cells. The prevalence and incidence of type-1 DM are increasing in the world.^4^ Subjects with type-1 DM had three times more mortality risk compared to the general population.^5^ The lifetime cost of a single patient with type-1 DM was estimated to be about USD 0.5 million.^6^

The long-term effects of uncontrolled DM are associated with many clinical problems. DM affects whole body systems and causes significant alterations in peri-articular and musculoskeletal system.^7^ Though musculoskeletal impairments are common in patients with DM but they have not been well recognized compared to cardiovascular complications.^7^

Shoulder pain is one of the prevailing complains among the diverse musculoskeletal diseases caused by DM.^8^ DM promotes the thickening of the joint capsule, causing adhesion of the humerus head to the glenohumeral cavity, the main cause of which is unknown. It has been reported that hyperglycemia may result in arthrofibrosis by speeding up glycation and irregular deposition of collagen in the connective tissues surrounding the joints.^7^ DM causes pain and limited joint mobility of one or both shoulders. It mitigates quality of life, and it also predisposes patients to have disability in daily activities.^7^-^9^

Earlier studies have shown high prevalence of shoulder disorders in patients having DM compared to controls.^7^,^10^-^13^ For example in a meta-analysis by Zreik et al.^10^ patients with DM were found to have five times more adhesive capsulitis than controls. Gutefeldt et al.^11^ reported 2-4 times more shoulder pain and stiffness in patients with DM than controls. Similarly, Raje et al.^12^ in their article concluded that diabetic patients had more shoulder pain and functional disability (SPFD) compared to controls. However, the majority of the formerly conducted studies included patients diagnosed with both types of DM or most commonly Type-2 of diabetes. Furthermore, most of the published studies, including Type-1 DM, have been conducted in US, Europe, and Australia and there is paucity of data in the Asian countries especially in Pakistan. The aim of the current study was to determine the prevalence of SPFD in patients with Type-1 DM and to explore the impact of duration of the disease, age and gender on SPFD.

## METHODS

A cross-sectional survey was conducted on previously diagnosed patients with Type-1 DM between April 2019 and March 2020. Data was collected from six hospitals including three tertiary care hospitals of Islamabad and Rawalpindi. The study was approved by the ethical review committee of Margalla Institute of Health Sciences Rawalpindi (Ref. No. AM/60/19, dated April 30, 2019). Three hundred and twenty-eight patients with 18 to 35 years of age and who were diagnosed with Type-1 DM were included in the study through convenience sampling technique. Patients with recent accident or trauma of shoulder joint, inflammatory arthropathy, bone and nerve pathologies, who were comatose or mentally ill and those whose occupation included repetitive shoulder activities were excluded. All the patients provided a written informed consent.

Data was collected by using demographic sheet and Shoulder Pain and Disability Index (SPADI). SPADI is a 13-items questionnaire designed to evaluate SPFD.^14^,^15^ It consists of two subscales, pain having 5 items and disability having 8 items. Each item score varies from 0 to 10. The total scores of SPADI ranges from 0 to 100. The higher scores indicate greater impairments. SPADI has shown good psychometric properties.^14^,^15^

### Sample size

Using 95% confidence interval, 5% error of measurement and prevalence rate of 30.57% reported by an earlier study,^16^ 327 participants were required.

### Data Analysis

Data analysis was performed using SPSS v.21. Mean and percentages were calculated to describe the results. Point-biserial and Pearson correlation coefficients were calculated to find out the correlation between the variables. Independent t-test was used to determine difference in the SPADI mean scores between the female and male patients. Five percent significance level was used for all tests.

## RESULTS

Three hundred and twenty-eight questionnaires were distributed to Type-1 diabetic patients. All of these were returned. Participants characteristics are shown in Table-I.

**Table-I:** Participants Characteristics (N = 328).

Variables		Mean	Standard deviation
Age (Years)		29.45	± 5.10
Duration of Diabetes (Years)		7.74	± 5.26

		**Frequency**	**Percentage**

Gender	Males	127	38.72%
	Females	201	61.28%
Hypertension		57	17.38%
Cardiovascular disease		10	3.05%
Generalized body pain		87	26.52%
Eye sight weakness		15	4.57%
Tingling/Numbness		34	10.36%
Polyuria		14	4.27%
Fatigue		16	4.88%

Out of 328 Type-1 diabetic patients, 281 (85.7%) patients were affected by SPFD. The mean SPADI score was 40.28±23.15. The SPFD was found significantly correlated to age, duration of the disease and gender (Table-II). It showed that increasing age and greater duration of the disease increases the SPFD.

**Table-II:** Association of the shoulder pain and disability to age, duration of diabetes mellitus and gender.

Variables	Shoulder pain and disability	

	r	P-value
Age (years)	0.332	< 0.001
Duration of Diabetes Mellitus (years)	0.154	0.005
Gender	0.171	0.002

A significant difference was found in the SPADI mean scores between female and male patients (female patients = 43.42±22.80, male patients = 35.31±22.91, p = 0.002). The results showed that female is at more risk for developing SPFD.

## DISCUSSION

This study highlights significantly high prevalence of SPFD in patients with Type-1 DM. This finding is consistent with the outcomes of earlier studies.^17^,^18^ However, it is slightly higher compared to the findings of Shah et al. and Ahmad et al.^19^,^20^ The prevalence rate of SPFD in the current study also varies with the findings of the other studies (24.9 – 41.3%).^16^,^21^-^23^ This might be due to the reason that in the current study the prevalence was reported about symptoms i.e., pain and disability rather than specific diagnosis (e.g., adhesive capsulitis) reported by these studies.

A recent study in Sweden reported shoulder pain and stiffness in 44% and 49% of diabetic patients respectively.^24^ The difference may be due to the questionnaires used for collecting data. In the Swedish study the data was collected by asking a single question about each pain and stiffness i.e., “do you have pain/stiffness in your shoulder joint”. Where as in the current study, the data was collected by using a reliable and valid questionnaire called SPADI which explored pain and disability by asking 13 different questions about these symptoms while performing different functional activities.

A significant correlation was found between SPFD and age of the patients. This was in line with the results of previous studies which further strengthens the notion that pain and disability at shoulder joint increases with increasing age in patients with Type-1 DM.^16^,^22^,^25^ The present study also found a significant correlation between SPFD and duration of DM. Previous studies results were comparable to these findings.^11^,^12^,^16^,^22^,^25^ It reflects that as the duration of DM increase shoulder pain disability increases as well.

The present study found that females with Type-1 DM were substantially associated with increased pain and disability at shoulder joint than males which is consistent with the findings of earlier studies.^11^,^16^,^18^,^20^,^23^

As the number of patients with Type-1 DM will increase, it is likely that shoulder pain and disability will become the most common complaint in these patients visiting diabetic clinics. Therefore, it would be more appropriate to include musculoskeletal examination of shoulder joint in the routine screening procedures in patients having Type-1 DM to prevent functional disability.

### Limitations of the study

As the study design was cross sectional therefore cannot give casual answers about underlying mechanism and determine changes over time. The results of current study cannot be generalized to a larger population due to small sample size.

## CONCLUSION

The prevalence of SPFD was found substantially high in Type-1 diabetic patients. Increasing age, longer duration of DM and female gender have significant association with pain and disability of shoulder joint. These findings suggest that there is a need to focus on periodical musculoskeletal examination of shoulder during follow-up and to develop strategies for rehabilitation in patients with Type-1 DM both for clinicians and researchers.

### Authors’ Contribution:

**MNF:** Concept, design, literature review, analysis and interpretation of data, writing of manuscript, critical revision of the article for important intellectual content. Responsible and accountable for the accuracy or integrity of the work.

**AM:** Concept, design, literature review, data collection, data analysis, writing of manuscript.

**FA:** Literature review, analysis and interpretation of data, writing of manuscript.

**JS:** Concept, design, data collection, writing of manuscript.

All authors have read and approved the manuscript.

## References

[1] Punthakee Z, Goldenberg R, Katz P (2018). Definition, Classification and Diagnosis of Diabetes, Prediabetes and Metabolic Syndrome. Can J Diabetes.

[2] Saeedi P, Petersohn I, Salpea P, Malanda B, Karuranga S, Unwin N (2019). Global and regional diabetes prevalence estimates for 2019 and projections for 2030 and 2045:Results from the International Diabetes Federation Diabetes Atlas, 9(th) edition. Diabetes Res Clin Pract.

[3] Akhtar S, Nasir JA, Abbas T, Sarwar A (2019). Diabetes in Pakistan:A systematic review and meta-analysis. Pak J Med Sci.

[4] Mobasseri M, Shirmohammadi M, Amiri T, Vahed N, Hosseini Fard H, Ghojazadeh M (2020). Prevalence and incidence of type 1 diabetes in the world:a systematic review and meta-analysis. Health Promot Perspect.

[5] Morgan E, Black CR, Abid N, Cardwell CR, McCance DR, Patterson CC (2018). Mortality in type 1 diabetes diagnosed in childhood in Northern Ireland during 1989-2012:A population-based cohort study. Pediatr Diabetes.

[6] Sussman M, Benner J, Haller MJ, Rewers M, Griffiths R (2020). Estimated Lifetime Economic Burden of Type 1 Diabetes. Diabetes Technol Ther.

[7] Hsu CL, Sheu WH (2016). Diabetes and shoulder disorders. J Diabetes Investig.

[8] Sozen T, Basaran N, Tınazli M, Ozisik L (2018). Musculoskeletal problems in diabetes mellitus. Eur J Rheumatol.

[9] Askari S, Imran N, Fawwad A, Butt A, Riaz M, Naseem R (2020). Health-related quality of life of Pakistani adolescents with type 1 diabetes and their parents. Int J Diabetes Dev Ctries.

[10] Zreik NH, Malik RA, Charalambous CP (2016). Adhesive capsulitis of the shoulder and diabetes:A meta-analysis of prevalence. Muscles Ligaments Tendons J.

[11] Gutefeldt K, Hedman CA, Thyberg ISM, Bachrach-Lindstrom M, Arnqvist HJ, Spangeus A (2019). Upper extremity impairments in type 1 diabetes with long duration;common problems with great impact on daily life. Disabil Rehabil.

[12] Raje YR, Cracknell G, Davoren PM (2015). Frequency of hand and shoulder symptoms in patients with type 1 diabetes. Diabet Med.

[13] Singla R, Gupta Y, Kalra S (2015). Musculoskeletal effects of diabetes mellitus. J Pak Med Assoc.

[14] Roach KE, Budiman-Mak E, Songsiridej N, Lertratanakul Y (1991). Development of a shoulder pain and disability index. Arthritis Care Res.

[15] MacDermid JC, Solomon P, Prkachin K (2006). The Shoulder Pain and Disability Index demonstrates factor, construct and longitudinal validity. BMC Musculoskelet Disord.

[16] Larkin ME, Barnie A, Braffett BH, Cleary PA, Diminick L, Harth J (2014). Musculoskeletal complications in type 1 diabetes. Diabetes Care.

[17] Holte KB, Juel NG, Brox JI, Hanssen KF, Fosmark DS, Sell DR (2017). Hand, shoulder and back stiffness in long-term type 1 diabetes;cross-sectional association with skin collagen advanced glycation end-products. The Dialong study. J Diabetes Complications.

[18] Juel NG, Brox JI, Brunborg C, Holte KB, Berg TJ (2017). Very High Prevalence of Frozen Shoulder in Patients With Type 1 Diabetes of ≥45 Years'Duration:The Dialong Shoulder Study. Arch Phys Med Rehabil.

[19] Shah KM, Clark BR, McGill JB, Mueller MJ (2015). Upper extremity impairments, pain and disability in patients with diabetes mellitus. Physiotherapy.

[20] Ahmad Q, Yaseen I, Sattar R, Abbas U, Nawaz U (2020). Prevalence of frozen shoulder among patients with diabetes:a single center experience from Karachi, Pakistan. Rawal Medical J.

[21] Doria C, Mosele GR, Badessi F, Puddu L, Caggiari G (2017). Shoulder Adhesive Capsulitis in Type 1 Diabetes Mellitus:A Cross-Sectional Study on 943 Cases in Sardinian People. Joints.

[22] Ahmad S, Rafi MS, Siddiqui IA, Hamidi K, Faruq NM (2012). The frequency of adhesive capsulitis in diabetes mellitus patients. Pak J Rehabilit.

[23] Inayat F, Ali NS, Shahid H, Younus F (2017). Prevalence and Determinants of Frozen Shoulder in Patients with Diabetes:A Single Center Experience from Pakistan. Cureus.

[24] Gutefeldt K, Lundstedt S, Thyberg ISM, Bachrach-Lindström M, Arnqvist HJ, Spangeus A (2020). Clinical Examination and Self-Reported Upper Extremity Impairments in Patients with Long-Standing Type 1 Diabetes Mellitus. J Diabetes Res.

[25] Arkkila PE, Gautier JF (2003). Musculoskeletal disorders in diabetes mellitus:an update. Best Pract Res Clin Rheumatol.

